# Investigating the Utility of Explainable Artificial Intelligence for Neuroimaging‐Based Dementia Diagnosis and Prognosis

**DOI:** 10.1002/hbm.70456

**Published:** 2026-02-02

**Authors:** Sophie A. Martin, An Zhao, Jiongqi Qu, Phoebe Imms, Andrei Irimia, Frederik Barkhof, James H. Cole, Alzheimer's Disease Neuroimaging Initiative

**Affiliations:** ^1^ UCL Hawkes Institute University College London London UK; ^2^ UCL Queen Square Institute of Neurology University College London London UK; ^3^ Leonard Davis School of Gerontology, University of Southern California Los Angeles California USA; ^4^ Department of Biomedical Engineering, Viterbi School of Engineering, Corwin D. Denney Research Centre University of Southern California Los Angeles California USA; ^5^ Department of Quantitative & Computational Biology, Dana and David Dornsife College of Arts & Sciences University of Southern California Los Angeles California USA; ^6^ Centre for Healthy Brain Aging, Institute of Psychiatry, Psychology and Neuroscience King's College London London UK; ^7^ Department of Radiology and Nuclear Medicine Amsterdam University Medical Centre Amsterdam the Netherlands

**Keywords:** dementia, diagnosis, explainable artificial intelligence, magnetic resonance imaging, neuroimaging, prognosis, vision transformers

## Abstract

Artificial intelligence and neuroimaging enable accurate dementia prediction but often involve ‘black box’ models that can be difficult to trust. Explainable artificial intelligence (XAI) aims to provide insights into the model's decisions; however, choosing the most appropriate method is non‐trivial and often context‐specific. We used T1‐weighted MRI to train models on two tasks: Alzheimer's disease (AD) classification (diagnosis) and predicting conversion from mild‐cognitive impairment (MCI) to all‐cause dementia (prognosis). We applied eleven XAI methods across two popular image classification architectures, producing visualisations of the most salient regions. We also propose a framework for interpreting explanations produced by different XAI methods and predictive models. Models achieved balanced accuracies of 81% and 67% for diagnosis and prognosis. XAI outputs highlighted brain regions relevant to AD with strong convergence across gradient‐based techniques. LIME produced explanations that were most similar across architectures. Mean saliency enhanced MCI prognosis prediction when included as an additional input feature. XAI can be used to verify that models are utilising relevant features and to generate valuable measures for further analysis.

## Graphical abstract

Artificial intelligence has the potential to enhance clinical decision making, but complex black box models can be difficult to interpret and trust. Explainable artificial intelligence methods aim to open the black box by highlighting which features are driving the model's prediction.

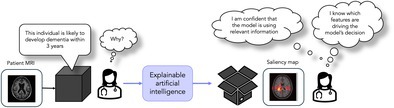

## 1 Introduction

Recent advancements in disease‐modifying therapies highlight the growing need for effective strategies to identify individuals at risk of dementia. Traditional diagnostic methods rely on brain imaging, cognitive performance assessments and long‐term observations of behavioural patterns. However, the increasing availability of large research datasets has enabled the use of artificial intelligence (AI) for diagnostic and prognostic modelling, with AI models demonstrating comparable and, in some cases, superior accuracy to traditional methods (Qiu et al. 2022; Xue et al. 2024; Martin, Townend, et al. 2023; Borchert et al. 2023).

To facilitate the integration of AI tools in clinical practice, it is essential to ensure that models are safe, robust and trustworthy. Explainable artificial intelligence (XAI) describes a set of techniques designed to provide reasoning for model decisions, allowing users to verify that the models are using relevant information and to detect potential biases. However, as the number of XAI methods continues to grow, determining the most appropriate technique has become challenging (Hedström et al. 2024; Adebayo et al. 2018; Das and Rad 2020; Miró‐Nicolau et al. 2024). Some studies have criticised XAI methods for their lack of robustness to noise and poor sensitivity to class‐specific features (Adebayo et al. 2018; Serrano and Smith 2019; Alvarez‐Melis and Jaakkola 2018). It is also difficult to validate XAI outputs in the absence of a ground‐truth, which is often the case for dementia studies due to the complex and heterogeneous nature of the underlying pathologies.

**FIGURE 1:**
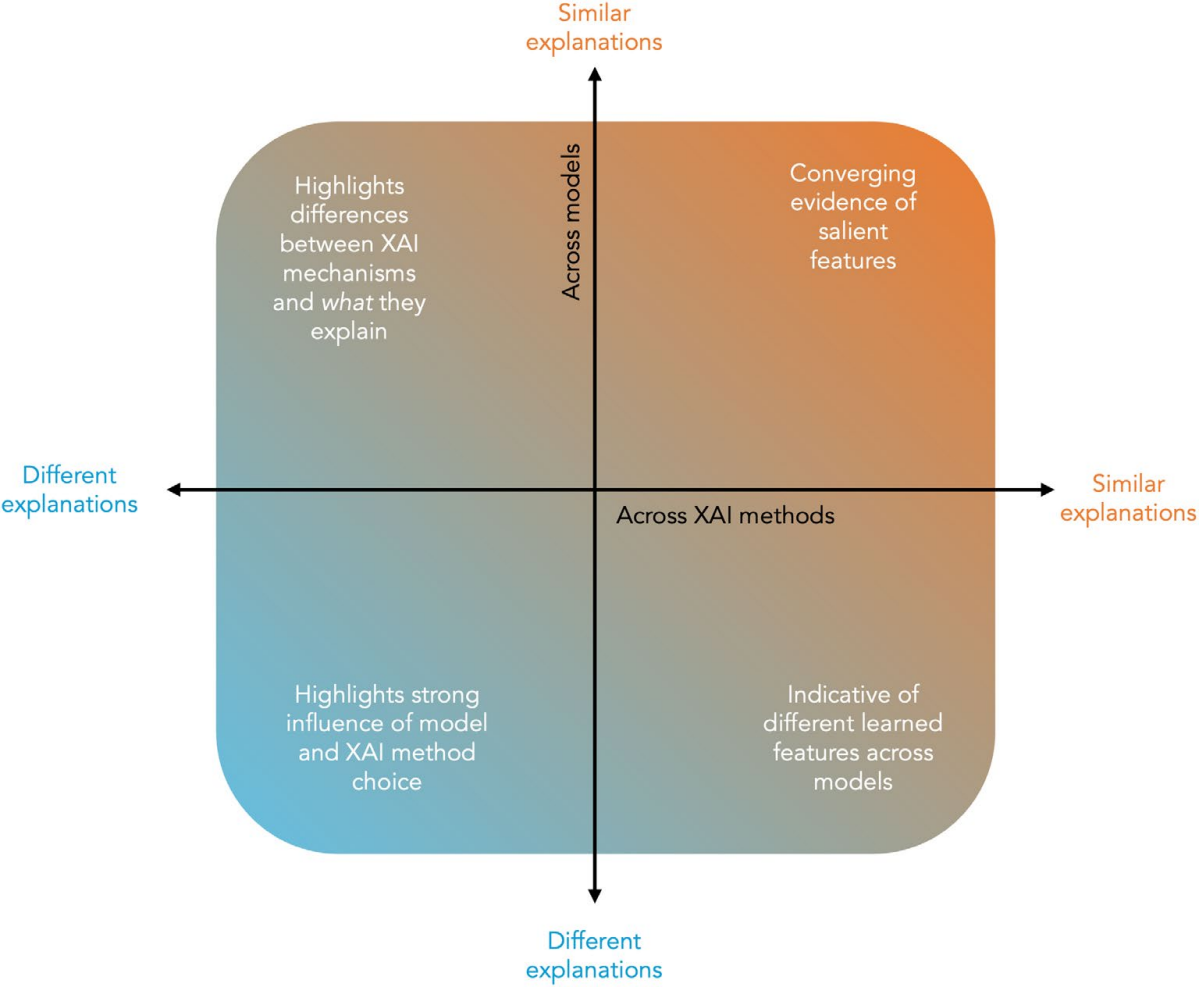
Overview of a framework summarising how to interpret XAI outputs, where different XAI methods act as ‘reviewers’ of the ‘evidence’ produced by different predictive models. If explanations are similar across multiple techniques and predictive models, this strengthens the validity of identified salient features. If explanations differ across XAI methods but not across models, then this highlights differences in the mechanisms of XAI approaches and *what* they explain. If explanations differ across predictive models but not across XAI methods, then this likely reflects differences in the learned features. If explanations vary both across models and XAI methods, it indicates a significant model‐method interplay. Systematic evaluation of this interplay in future work will be important, and to have accurate interpretation, researchers will need to account for this variability, or identify which results are most appropriate based on the predictive task and the purpose of the explanation.

In this work, we applied several existing XAI techniques to produce dementia‐related saliency maps (also known as ‘heatmaps’). We also present a framework for interpreting and comparing outputs: where XAI methods can be considered as ‘reviewers’, each assessing the importance of input features, and different predictive models extract their own ‘evidence’ of the target class. Similar explanations across multiple models can strengthen the validity of salient features while discrepancies can reveal fundamental differences between the models or highlight the relative advantages or disadvantages of certain XAI techniques. This is depicted in Figure 1. Additionally, to explore whether model explanations capture diagnostically useful information we assessed the utility of saliency map‐derived features in a downstream task of predicting future conversion to dementia.

Studies exploring deep learning for dementia prediction have reported classification accuracies between 70% and 99% for distinguishing Alzheimer's disease (AD) patients from healthy controls and 60%–90% for predicting mild‐cognitive impairment (MCI) conversion (Martin, Townend, et al. 2023; Borchert et al. 2023). However, many did not assess model explanations. Those that do often rely on a single XAI method, with comparisons of multiple methods remaining relatively underexplored in dementia applications (Martin, Townend, et al. 2023). Bloch and colleagues conducted a systematic evaluation of deep learning and classical models for dementia diagnosis (Bloch and Friedrich 2024) demonstrating comparable performance between models based on volumetric features (89.6% balanced accuracy (BACC)) and whole‐brain neural networks (83.6% BACC). They compared the most influential brain regions identified by three XAI methods across classical and deep learning approaches. Liu and colleagues applied backpropagation to a 3D CNN trained to distinguish between healthy controls, MCI and AD. In both cases, the authors found salient regions that are commonly linked to dementia, such as the left hippocampus. However, Bloch and colleagues also noted that deep learning models were more likely to rely on regions that are less commonly associated with AD (Bloch and Friedrich 2024). With the high variability of XAI outputs across methods and the lack of consensus on the most reliable approach, it is difficult to confirm the validity of new findings, and frameworks for quantitative evaluation of XAI outputs have yet to be popularised.

The emergence of vision transformers (ViT) provides an attractive alternative to convolutional neural networks (CNNs) for analysing neuroimaging data, with ViTs demonstrating superior predictive performance in many image‐classification tasks (Dosovitskiy et al. 2020). Moreover, ViTs are proposed to be inherently more interpretable (Park and Kim 2022; Raghu et al. 2021); their built‐in attention mechanism explicitly captures relationships between all input regions and provides a direct way to understand their influence on the model's prediction. On the other hand, CNNs require post hoc methods to generate explanations, as their use of abstract local features and their hierarchical structure make it difficult to trace predictions back to specific regions in the input space. However, to achieve their performance advantages, ViTs often require training on million‐scale datasets which are scarce in the medical imaging domain (Khan et al. 2021). To overcome this limitation of ViTs, researchers have used so‐called ‘transfer learning’ which uses models that are pretrained on larger, non‐medical datasets and finetuned on smaller task‐specific datasets (Usman et al. 2022; Gani et al. 2022; Malpure et al. 2021).

In this work, we applied transfer learning to finetune CNNs and ViTs for dementia classification and compared their diagnostic and prognostic performance. Eleven post hoc XAI methods were applied to generate group‐level saliency maps and investigate the neuroanatomical patterns associated with dementia classification. Additionally, XAI outputs were incorporated into a downstream classification model for MCI prognosis to explore whether they can increase predictive power.

## 2 Methods

**FIGURE 2:**
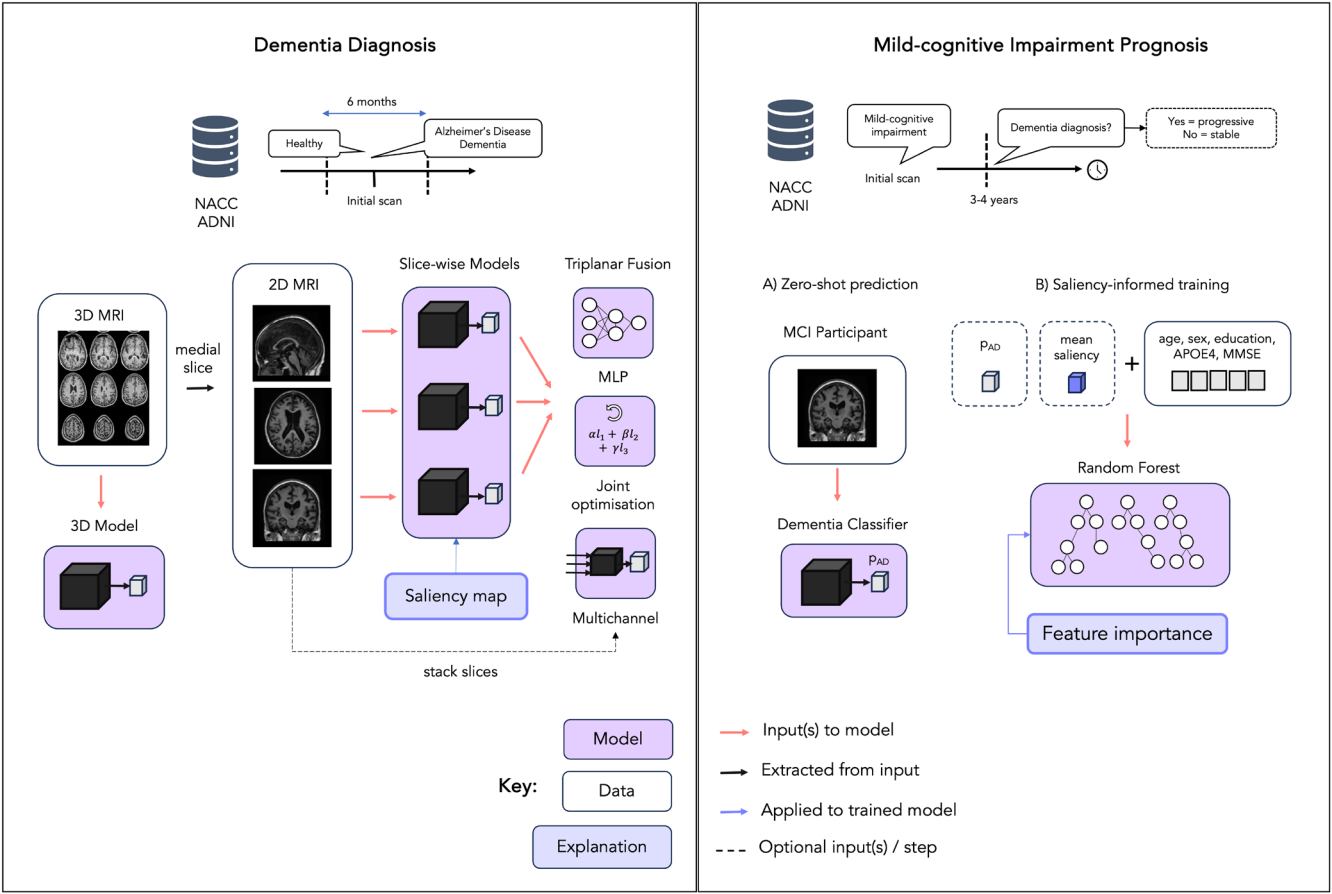
Study overview. Data from NACC was used to train and test the models. Labels were assigned using clinical diagnoses at visits within 6 months (diagnosis) or between 3 to 4 years (prognosis) from the date of the MRI scan. Data from ADNI was used as an external test set. Labels provided at the time of scan were used for diagnosis or assigned based on the same 3 to 4‐year window for MCI prognosis. For dementia classification, we applied transfer learning to finetune models on an axial, sagittal and coronal medial slice. We also trained a 3D model (3D ResNet) and explored the use of three triplanar fusion strategies to combine information from each plane as an intermediary approach: (i) adding a MLP layer to each output, (ii) using a weighted loss to jointly optimise each network, (iii) stacking slices from plane along the channel dimension. Then we applied eleven explanation methods to the 2D models to generate dementia‐related saliency maps. For MCI prognosis: (A) We computed the performance of models trained on AD dementia classification for predicting conversion from MCI to all‐cause dementia. (B) We explored the utility of including mean saliency and AD probability, p_AD,_ as features in a Random Forest model trained and tested on MCI prognosis. MCI = mild‐cognitive impairment, MMSE = mini‐mental state examination, MLP = multilayer perceptron.

The study overview is depicted in Figure 2.

### 2.1 Dataset

We used baseline demographic, clinical and T1‐weighted magnetic resonance imaging (MRI) data collated from the National Alzheimer's Coordinating Centre (NACC) and the Alzheimer's Disease Neuroimaging Initiative (ADNI). Models were trained using 90% of the NACC sample and tested on the remaining 10% (split at the participant level). ADNI data were used as an external test set to assess generalisability. A summary of the demographics of each dataset is given in Table 1.

**TABLE 1:** NACC and ADNI dataset characteristics for training and test samples. Due to missing data for certain participants across the demographic features, the sample size, *n*, is given for each feature and subgroup where participants with missing data have been excluded. The mean and standard deviation (or where appropriate, %) is given for each group label (AD, CN, sMCI, pMCI).

Feature	Training sample			Test sample					
	Diagnosis			Diagnosis			Prognosis		
	Label	*n*	Mean ± std./%	Label	*n*	Mean ± std./%	Label	*n*	Mean ± std./%
NACC									
Age	All AD CN	2754 646 2108	69.5 ± 11.0 73.5 ± 9.68 68.3 ± 11.1	All AD CN	306 63 243	69.0 ± 10.3 72.0 ± 8.95 68.2 ± 10.5	All pMCI sMCI	350 176 174	73.7 ± 7.88 74.7 ± 7.84 72.6 ± 7.80
Sex (% female)	All AD CN	2754 646 2108	62.6 51.2 66.1	All AD CN	306 63 243	63.4 55.6 65.4	All pMCI sMCI	350 176 174	42.9 41.5 44.3
Education (years)	All AD CN	2750 645 2105	15.8 ± 2.98 15.0 ± 3.47 16.0 ± 2.76	All AD CN	306 63 243	15.6 ± 3.18 14.2 ± 3.71 15.9 ± 2.93	All pMCI sMCI	349 176 173	15.6 ± 3.16 15.4 ± 3.11 15.8 ± 3.20
APOE4 (% positive)	All AD CN	2341 572 1769	40.4 62.9 33.1	All AD CN	262 56 206	39.3 55.4 35.0	All pMCI sMCI	343 172 171	52.8 55.2 50.3
MMSE	All AD CN	1278 391 887	26.4 ± 4.84 20.9 ± 5.27 28.9 ± 1.41	All AD CN	139 39 100	26.3 ± 5.20 19.9 ± 5.57 28.8 ± 1.87	All pMCI sMCI	227 128 99	26.4 ± 2.69 25.7 ± 3.11 27.3 ± 3.20
ADNI (External test sample)									
Age				All AD CN	130 36 94	72.8 ± 7.45 76.3 ± 8.11 71.5 ± 6.75	All pMCI sMCI	354 86 268	75.1 ± 7.81 77.4 ± 7.29 74.3 ± 7.83
Sex (% female)				All AD CN	130 36 94	53.1 47.2 55.3	All pMCI sMCI	354 86 268	40.4 45.3 38.8
Education (years)				All AD CN	122 32 90	16.5 ± 2.25 15.4 ± 2.20 16.9 ± 2.13	All pMCI sMCI	354 86 268	16.0 ± 2.73 15.8 ± 2.71 16.1 ± 2.74
APOE4 (% positive)				All AD CN	107 32 75	30.0 62.5 25.3	All pMCI sMCI	351 86 265	30.7 51.2 35.8
MMSE				All AD CN	122 32 90	27.4 ± 2.87 23.5 ± 2.16 28.8 ± 1.50	All pMCI sMCI	354 86 268	28.0 ± 1.67 27.7 ± 1.75 28.1 ± 1.63

Abbreviations: AD = Alzheimer's disease; CN = cognitively normal, (s/p) MCI = (stable/progressive) mild‐cognitive impairment; MMSE = mini‐mental state examination.

#### 2.1.1 NACC

The NACC is a centralized data repository on Alzheimer's disease and related dementias. It contains data from over 50,000 participants, ranging from cognitively normal individuals to those with mild cognitive impairment or dementia symptoms, with longitudinal scans and follow‐up information for over 17,000 participants. Our analysis contains data from 25 centres and utilises clinical information from standardised visits conducted between September 2005 and May 2022 (June 2022 data freeze #58). Diagnoses are consensus‐based and rely primarily on clinical, cognitive and functional evaluations. Imaging data is not routinely used, as it depends on availability and site‐specific protocols. The cohort used in this study consisted of 3410 participants (2351 CN, 709 AD, 174 stable MCI, 176 progressive MCI). Class labels were defined by the reported diagnosis at the closest visit within 6 months of the baseline scan date. For the prognostic task of predicting conversion from MCI to dementia, we assigned stable and progressive MCI labels using baseline and follow‐up diagnoses. Participants who were MCI at baseline and later received a dementia diagnosis within a 3 to 4‐year window were labelled as progressive MCI (pMCI). Participants who were MCI at baseline and receive an additional MCI diagnosis after 3 years or more were treated as stable MCI (sMCI).

#### 2.1.2 ADNI

The ADNI was launched in 2003 and hosts a large dementia dataset curated from numerous research centres across the United States and Canada. The original goal was to combine imaging data, clinical and neuropsychological assessment and other biomarkers to measure the progression of MCI and early AD. Current goals include validating biomarkers for clinical trials, improving the generalizability of ADNI data by increasing diversity in the participant cohort, and providing data on the diagnosis and progression of AD. ADNI contains several imaging modalities and clinical assessments used for diagnoses such as CN, MCI and AD. ADNI incorporates rigorous image quality control procedures, including standardized protocols for MRI acquisition, preprocessing and quality assurance to ensure consistency and reliability across sites. Participants receive a baseline visit and clinical assessment, followed by regular follow‐up visits to produce a structured longitudinal database of dementia trajectories. Diagnostic labels are assigned based on expert consensus and all available participant data. In this study, we used T1‐weighted MRI and diagnostic labels from a subset of 484 participants across ADNI‐1, ADNI‐2 and ADNI‐3 cohorts (94 CN, 36 AD, 268 sMCI, 86 pMCI). Participants with a diagnosis of MCI who do not go on to receive a diagnosis of AD at any follow‐up visits for at least 3 years were labelled as sMCI. To encourage consistency with the criteria used for NACC participants, we assigned stable and progressive MCI labels using a 3 to 4‐year window. For sMCI, MRI scans 3 years before the last MCI diagnosis were used. For the pMCI group, we extracted MRI scans within a 3 to 4‐year window before the first diagnosis of AD. Importantly, while the pMCI NACC cohort were labelled based on all‐cause dementia diagnoses, ADNI patients all progressed to dementia of the Alzheimer's type.

### 2.2 Data Processing and Quality Control

For each dataset, scans were minimally pre‐processed by affine registration to the asymmetric MNI‐152 1 × 1 × 1 mm resolution template using EasyReg (Iglesias 2023), padded to square (or cube) and resized, followed by intensity scaling to the range 0 to 1 and normalisation using the image‐level mean intensity and standard deviation across the training cohort. No skull stripping was applied. For 2D models, mid‐stack slices were extracted from each registered volume along three axes (axial, sagittal, coronal) and were resized to 224 × 224 mm. For the 3D model, volumes were resized to 96 × 96 × 96 mm. NACC imaging data is voluntarily provided by individual ADRCs and is not subject to a standardised imaging protocol or quality control. Therefore, these scans were visually assessed and passed through an automated image quality toolbox, MRIqc (Esteban et al. 2017). Scans that failed either visual assessment or produced outliers in terms of MRIQC‐derived image quality metrics (IQMs)—contrast, signal‐to‐noise and the coefficient of joint variation—were removed (*n* = 158). The NACC participant inclusion criteria and data quality control procedure are detailed in Supporting Information: Figure A1. We also computed IQMs for ADNI scans; however, since ADNI has its own acquisition and quality control procedures, we did not exclude scans based on these. Nonetheless, the IQMs highlight larger variability in image quality across the NACC dataset compared to ADNI, as shown in Supporting Information: Figure A2.

### 2.3 Model Training

#### 2.3.1 ResNet

The ‘residual network’ or ResNet model (He et al. 2015) is a type of CNN and a popular choice for image classification. ResNets consist of a series of convolutional layers, which are organized into blocks. They use residual connections between layers to give the model the flexibility to learn from previous layers and prevent issues such as vanishing gradients. The input image is passed sequentially through the network, allowing each convolutional layer to extract features at different resolutions via learned filters.

#### 2.3.2 Vision Transformers

ViTs (Dosovitskiy et al. 2020) are an extension of the original Transformer (Kindermans et al. 2019) network that was first developed for natural language processing, applied to image data. Unlike traditional CNNs, which process images using local filters, ViTs divide an image into patches, flatten them into one‐dimensional vectors and treat these patches as a sequence of tokens, analogous to words in text. These tokens are then processed by transformer layers, which use the so‐called ‘self‐attention’ mechanism to capture statistical dependencies between the patches and thus model complex relationships within the image. The output of the final transformer layer can be linearly transformed and used for classification or other tasks.

#### 2.3.3 Transfer Learning

Training ViTs from scratch remains a significant challenge, particularly in small dataset settings, where they often offer marginal or no improvements over conventional CNNs (Gani et al. 2022; Lee et al. 2021; Springenberg et al. 2023). However, ViTs have demonstrated superior performance compared to CNNs when pretrained on sufficiently large‐scale datasets (Dosovitskiy et al. 2020; Khan et al. 2021). As of this writing, publicly available pretrained 3D ViT models suitable for fine‐tuning on medical imaging datasets are limited. To address this, we leveraged pretrained 2D ViTs from the timm library (Wightman et al. 2023) which have outperformed CNNs in the natural image domain and developed a framework to finetune them using 2D slices extracted from 3D MRI volumes. We also applied this framework to timm's pretrained 2D ResNets to compare the predictive performance for dementia prediction tasks and explanations produced by post hoc methods across both architectures. To transfer learn from pretrained 2D models, we finetuned each model using mid‐stack axial, sagittal and coronal slices. All model weights were allowed to be updated during finetuning. Both models were initially trained on ImageNet‐1k (Deng et al. 2009) allowing the models to learn high‐level, generalisable patterns.

#### 2.3.4 Training Parameters, Tuning and Optimisation

Models were finetuned using data from NACC. To identify a suitable training configuration, we began by performing a hyperparameter search for the axial‐based ResNet and ViT, by varying the learning rate, dropout rate, learning rate scheduler and optimiser. Details on the hyperparameter search space are provided in Supporting Information: Table A5. The optimal parameters were chosen based on the area‐under‐the‐precision‐recall curve (AUPRC) using 20% of the training data for validation.

The fixed configuration was used to finetune the sagittal and coronal models and retrain the axial model using 90% of the training data. Early stopping was used to reduce overfitting with the remaining 10% of the training data (patience of 20, based on the AUPRC). Additionally, random data augmentations from the monai library were applied during training to encourage robustness (left–right flip, zoom, contrast, Gaussian noise and Gibbs noise). This produced finetuned models for each model architecture: ViT‐axial, ResNet‐axial, ViT‐coronal, ResNet‐coronal, ViT‐sagittal and ResNet‐sagittal.

#### 2.3.5 Triplanar Models

Many existing studies have shown that 3D models outperform their 2D counterparts due to the ability to leverage contextual information across the volume (Martin, Townend, et al. 2023; Ebrahimi and Luo 2021). This is particularly relevant for neuroimaging data, as biologically relevant information is often present throughout the brain volume, rather than a single slice (Ebrahimi and Luo 2021). Due to the limited availability of pretrained 3D ViT models, we have focused on models trained on 2D slices throughout this study. However, as a mitigation approach we also explored fusion strategies to combine information across three orthogonal slice views: axial, sagittal and coronal. First, we trained an ensemble model which combines the predictions of three independent single‐plane models in a fully connected feed‐forward multilayer perceptron (MLP), typically referred to as late fusion. A hyperparameter search was used to determine the optimal depth and number of layers of the MLP, with details provided in Supporting Information: Table A6. We also explored a stacking strategy, where the three views were stacked along the channel dimension, allowing information from each slice to be captured by a single 2D model. Thirdly, we implemented a joint optimisation strategy in which the weights of three single‐plane models are jointly learned via a weighted loss, L, given by:

$$L=\alpha_{\text{axial}}l_{\text{axial}}+\alpha_{\text{sagittal}}l_{\text{sagittal}}+\alpha_{\text{coronal}}l_{\text{coronal}}$$

where a are factors optimised during training using a binary cross‐entropy loss and training procedure described above. From a clinical perspective, this serves as a useful indicator of the relative advantages and disadvantages between slice‐wise and volumetric analysis.

#### 2.3.6 Performance Evaluation

The predictive performance of the models was evaluated using three classification metrics: BACC, the area‐under‐the‐receiver‐operator‐curve (AUROC), and AUPRC on the held‐out test set (diagnosis) and MCI participants (prognosis). The AUPRC was used for optimising models during hyperparameter tuning and early stopping as it focuses on the positive class (AD), which is important for imbalanced datasets (Saito and Rehmsmeier 2015). Five‐fold cross validation was also performed to assess the stability of the single‐slice models (diagnosis only). To benchmark the 2D models, we also trained and evaluated a 3D ResNet‐18 from scratch (using the monai library implementation (Monai 2020) and the same training configuration as the 2D models). To assess cross‐cohort generalisability, data from ADNI were used to test the trained models on the diagnostic and prognostic tasks.

### 2.4 Explainable Artificial Intelligence Methods

We primarily used the captum (Kokhlikyan et al. 2020) library to implement popular explanation methods for the two model architectures. This included model‐agnostic methods such as occlusion (Zeiler and Fergus 2014), Shapley additive explanations (SHAP) (Shapley 2016) and local interpretable model explanations (LIME) (Ribeiro et al. 2016). We also used this library to apply methods tailored for explaining neural networks such as guided and unguided backpropagation (Simonyan et al. 2014), Integrated Gradients (Sundararajan et al. 2017) (35)and InputXGradient (Qiu et al. 2022), guided and unguided gradient‐weighted class activation mapping (GradCAM) (Selvaraju et al. 2016) and layerwise relevance propagation (LRP) (Bach et al. 2015). These methods all rely on access to the gradients learned during training but vary in their mechanisms for propagating values into the input image space. However, some of these methods (e.g., GradCAM) cannot be applied to ViTs or ResNets naively because they were originally designed for CNNs. Therefore, we used an alternative implementation by Gildenblat and colleagues to generate GradCAM maps for ViTs and ResNets, which accounts for the different intermediate feature shapes so that they are compatible with the original algorithm (Gildenblat 2021). Similarly, to apply the LRP method to ResNets, care must be taken to account for skip connections, which are not in the original design or captum implementation. Therefore, we used code from Otsuki and colleagues (Otsuki et al. 2024) which uses relevance splitting to apply LRP to the ResNet architecture (minimally adapted for compatibility with timm models). The LRP method was not applied to ViTs; however, we used attention rollout to generate model‐specific explanations. Attention rollout iteratively multiplies the attention weights from all layers, effectively tracing how information is propagated from the input to model output. This provides explanations without requiring a separate post hoc mechanism. The saliency maps were thresholded based on their intensities to identify the most salient regions (top 10%) and reduce noise. We also applied a ReLU operation to focus on positive values and scaled each map by the maximum value so that the intensities ranged from 0 to 1.

#### 2.4.1 Comparing Model Explanations

Our work seeks to understand whether brain‐relevant features are being used by the model to inform predictions, identify potential learned biases and determine the utility of saliency‐derived features. First, we assessed the amount of saliency assigned to brain versus non‐brain regions across the different XAI methods as an approximate measure of brain specificity and initial quality check. This involved computing the proportion of non‐zero pixels that lie outside of the brain using the MNI152 brain mask.

To assess whether model explanations were specific to AD, we generated group‐level saliency maps by averaging the outputs across methods for different subgroups (true positives + false positives = ‘AD’, true negatives + false negatives = ‘CN’). These tests aimed to shed light on the variability and specificity of XAI outputs across different techniques. However, it is important to note that a true evaluation of their utility is difficult due to the lack of individual level ground‐truth references. Whilst group‐level saliency maps can be compared to known biologically relevant ROIs, individual‐level validation is more complex due to the heterogeneous atrophy patterns across the AD continuum, some of which can be captured by normative models (Verdi et al. 2023).

Additionally, a frequently reported limitation of XAI is its sensitivity to the underlying model despite the same input data. Some studies have quantified this sensitivity by randomizing layer weights and observing the effect on XAI outputs (Hedström et al. 2024; Adebayo et al. 2018). Here, we computed the Dice coefficient based on the most salient regions (top 10% intensity percentile) of the average ‘AD’ saliency map across the different models and methods. This provides an assessment of the agreement of the most salient features across the different methods and architectures. The Dice coefficient is given by:

$$\text{Dice}=2\frac{|X|\cap |Y|}{\left|X\right|+|Y|}$$

where $\left|X\right|$ and $\left|Y\right|$ correspond to binary maps and ∩ refers to their overlap.

#### 2.4.2 Prognostic Value of Model Explanations

To assess the predictive prognostic value of the model explanations we trained a Random Forest model to classify pMCI from sMCI, using mean saliency values from each participant. Firstly, we used our trained dementia classifier to obtain a ‘dementia’ saliency map for each MCI participant. To reduce noise, we removed non‐brain pixels using an MNI152 brain mask and applied ReLU and normalisation as before. The mean saliency was included as a feature alongside relevant clinical features (age, sex, education, APOE4 status, MMSE score) and optionally, the zero‐shot model‐predicted probability, p_AD_. For this analysis, we used 75% of MCI participants for training and the remaining 25% for testing. After removing participants with missing features, this gave a training sample of 164 and a test sample of 56 participants. Models were trained and tuned using the scikit‐learn 5‐fold grid‐search cross‐validation procedure. Hyperparameters and the search space used are provided in Supporting Information: Table A7.

We benchmarked the addition of saliency against two baseline models: one that used clinical and demographic features only and another with p_AD_ as a feature. To capture the variability in the trained models, we report the average and standard deviation across 25 repeats for each input configuration (various random seeds). Furthermore, once the optimal combination of saliency features and probabilities was found, we applied SHAP using the captum (Kokhlikyan et al. 2020) library to identify the influence of each feature.

## 3 Results

### 3.1 Classification Performance

**FIGURE 3:**
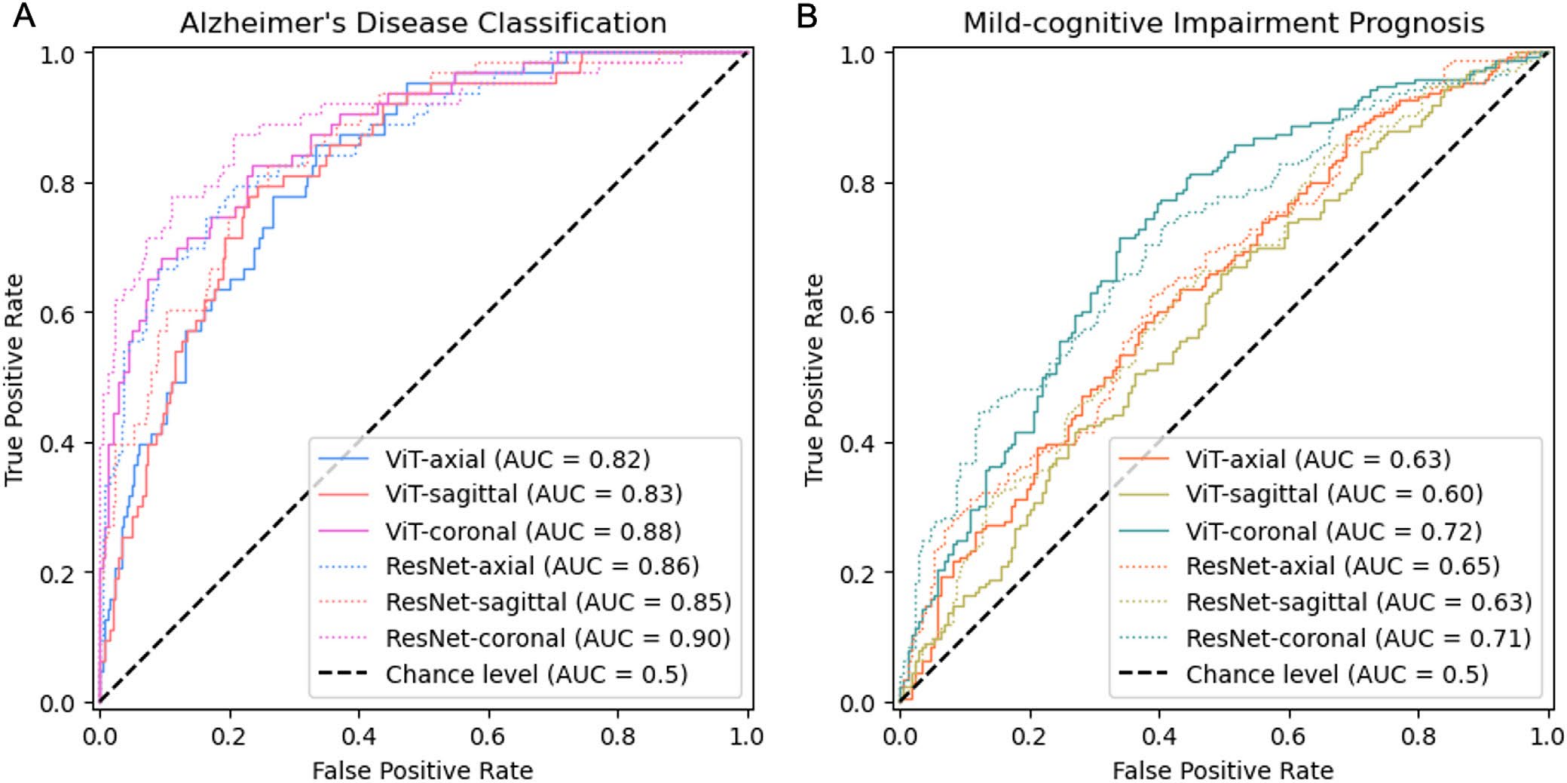
(A) Receiver‐operator curves for single‐slice ResNet and vision transformer models trained and tested on NACC data for dementia classification (AD versus CN). Colours correspond to the different slice views—sky‐blue: Axial, pink: Coronal, red: Sagittal. (B) Receiver‐operator curves for single‐slice ResNet and vision transformer models tested on MCI prognosis (sMCI versus pMCI). Colours correspond to the different slice views: Blue‐green = axial, orange = coronal, yellow = sagittal.

Overall, our models were able to classify between dementia patients and controls, or between pMCI and sMCI, though less accurately. Table 2 provides the test BACC, AUROC and AUPRC values on the held‐out test set for diagnosis, and MCI participants for prognosis. Figure 3a,b demonstrate the receiver‐operator curve (ROC) performance for the six ResNet and ViT models trained and tested on the NACC dataset, with AUC (area under curve) values shown in the legend for the diagnosis and prognosis tasks respectively. To summarise the 2D versus 3D classification accuracy, we report the difference between the 3D model and the single‐slice model with the highest BACC. For dementia classification, a BACC of 78.7% was obtained using a mid‐stack coronal ViT model compared to 83.4% with a fully 3D model. This difference between 2D and 3D models was even smaller (−3.6%) for MCI prognosis. Cross‐validation results are provided in Supporting Information: Table A8, demonstrating the stability of the diagnostic performance across five folds.

**TABLE 2:** Performance of single slice and triplanar models for two tasks, diagnosis and prognosis (zero‐shot) across both architectures in a held‐out test set from the NACC data.

NACC test performance													
		Diagnosis (AD (63) vs. CN (243))						Prognosis (sMCI (174) vs. pMCI (176))					
		Vision Transformer			ResNet			Vision Transformer			ResNet		
		BACC	AUROC	AUPRC	BACC	AUROC	AUPRC	BACC	AUROC	AUPRC	BACC	AUROC	AUPRC
Single slice	Axial	68.6	82.5	55.0	77.2	86.5	70.0	56.7	63.4	61.2	56.3	65.1	66.0
	Coronal	78.7	87.8	72.5	75.2	89.7	80.4	63.8	72.1	69.9	62.1	71.4	72.6
	Sagittal	65.9	82.6	54.3	68.8	85.4	65.9	56.1	59.6	58.5	56.2	63.2	61.5
Triplanar	Stacked	77.8	88.5	72.2	81.1	90.5	79.7	60.6	64.6	62.8	65.0	72.0	71.6
	Joint	78.6	89.1	73.2	80.7	91.7	82.2	63.6	70.4	69.9	67.0	73.4	73.1
	Late	74.7	87.6	67.9	78.5	90.5	80.2	61.8	70.3	67.3	67.3	72.6	72.2
	3D	—	—	—	83.4	89.5	82.1	—	—	—	65.7	72.7	73.0

Abbreviations: AD = Alzheimer's disease; AUPRC = area‐under‐the‐precision‐recall‐curve; AUROC = area‐under‐the‐receiver‐operator‐curve; BACC = balanced accuracy; CN = cognitively normal; (s/p) MCI = (stable/progressive) mild‐cognitive impairment.

#### 3.1.1 Triplanar Fusion

To leverage information from orthogonal views of the medial slice, we compared three strategies: stacked, joint and late fusion. Of the three methods, joint fusion led to the best AUPRC, most evident when the ResNet triplanar models were evaluated on prognosis. For this task, the joint model even exceeded the performance of the fully‐3D architecture.

#### 3.1.2 Cross‐Cohort Generalisability

To assess the generalisability of these results we also evaluated the cross‐cohort performance using data from ADNI (Table 3). The model achieved similar performance as in the NACC test set with optimal BACCs of 85.4% (difference +2.0%) and 67.3% (difference +2.1%) for diagnosis and prognosis, respectively.

**TABLE 3:** Cross‐cohort generalisability of NACC‐trained single slice and triplanar models for two tasks, diagnosis and prognosis across both architectures in a held‐out test set from the ADNI data.

ADNI test performance													
		Diagnosis (AD (36) vs. CN (94))						Prognosis (sMCI (286) vs. pMCI (86))					
		Vision transformer			ResNet			Vision transformer			ResNet		
		BACC	AUROC	AUPRC	BACC	AUROC	AUPRC	BACC	AUROC	AUPRC	BACC	AUROC	AUPRC
Single slice	Axial	75.8	84.4	66.8	70.2	84.6	69.1	60.9	64.0	36.6	65.7	68.1	40.9
	Coronal	80.3	90.0	85.1	74.3	93.2	86.3	65.4	71.7	44.8	58.4	72.6	44.0
	Sagittal	65.4	77.6	55.9	76.0	87.4	75.9	56.5	62.9	33.1	57.9	65.8	34.4
Triplanar	Stacked	78.1	87.9	80.6	81.7	91.3	85.9	65.1	69.4	41.1	62.8	72.6	45.6
	Joint	77.2	88.8	79.4	81.5	93.0	86.2	63.6	69.1	39.2	67.9	75.6	48.5
	Late	78.9	89.1	82.2	79.6	91.1	84.1	64.5	70.6	43.1	64.8	73.3	48.1
	3D	—	—	—	85.4	94.6	89.6	—	—	—	69.4	73.4	46.1

Abbreviations: AD = Alzheimer's disease; AUPRC = area‐under‐the‐precision‐recall‐curve; AUROC = area‐under‐the‐receiver‐operator‐curve; BACC = balanced accuracy; CN = cognitively normal; (s/p) MCI = (stable/progressive) mild‐cognitive impairment.

### 3.2 Saliency Maps for Dementia Diagnosis

**FIGURE 4:**
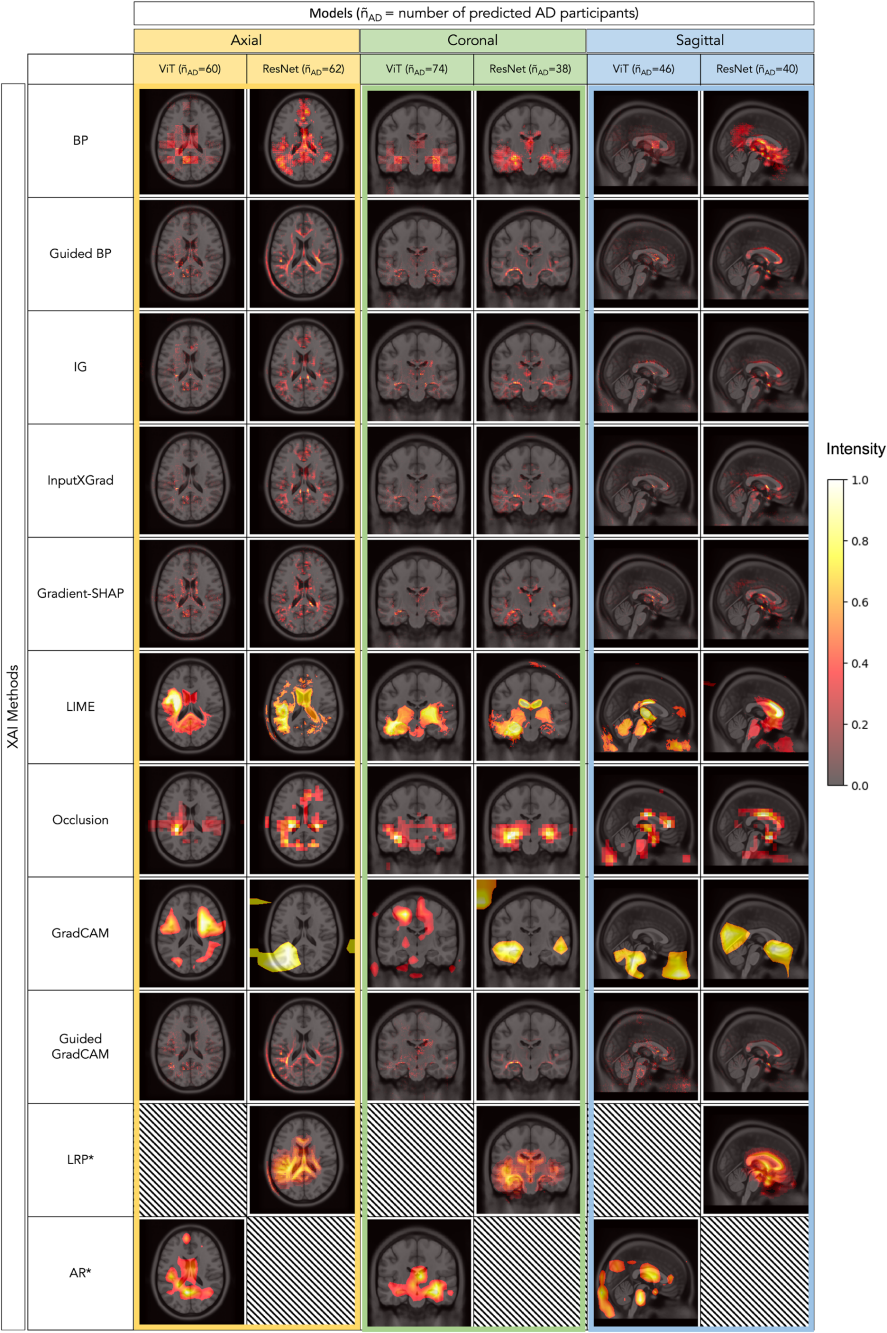
Top 10% of salient pixels for saliency maps generated by eleven XAI methods (averaged over positive ‘AD’ model predictions (TP + FP) with the corresponding number of participants, ñ_AD_, in brackets). ‐A = axial (yellow); AR = attention rollout; BP = backpropagation; ‐C = coronal (green); CAM = class activation mapping; IG = integrated gradients; InputXGrad = InputxGradient; LIME = local interpretable model explanations; LRP = layer wise relevance propagation; ‐S = sagittal (blue); SHAP = Shapley additive explanations; ViT = vision transformer. * Denotes methods that were not implementable for both architectures.

We applied eleven distinct explanation techniques to identify which regions the model used to inform its prediction. First, we explored this on a group level by producing average ‘AD’ saliency maps based on the model's true and false positive predictions, which were thresholded to visualise the most salient pixels (Figure 4). As a high‐level verification, we assessed whether the models learned to focus on the brain, both visually and by computing the proportion of non‐brain saliency (Supporting Information: Figure A4). This was confirmed as the most salient pixels fell predominantly within the brain mask. Across the methods, we observed high saliency in regions such as the ventricles, hippocampus, corpus callosum and cingulate gyrus.

#### 3.2.1 Do Explanations Vary Depending on Network Architecture?

**FIGURE 5:**
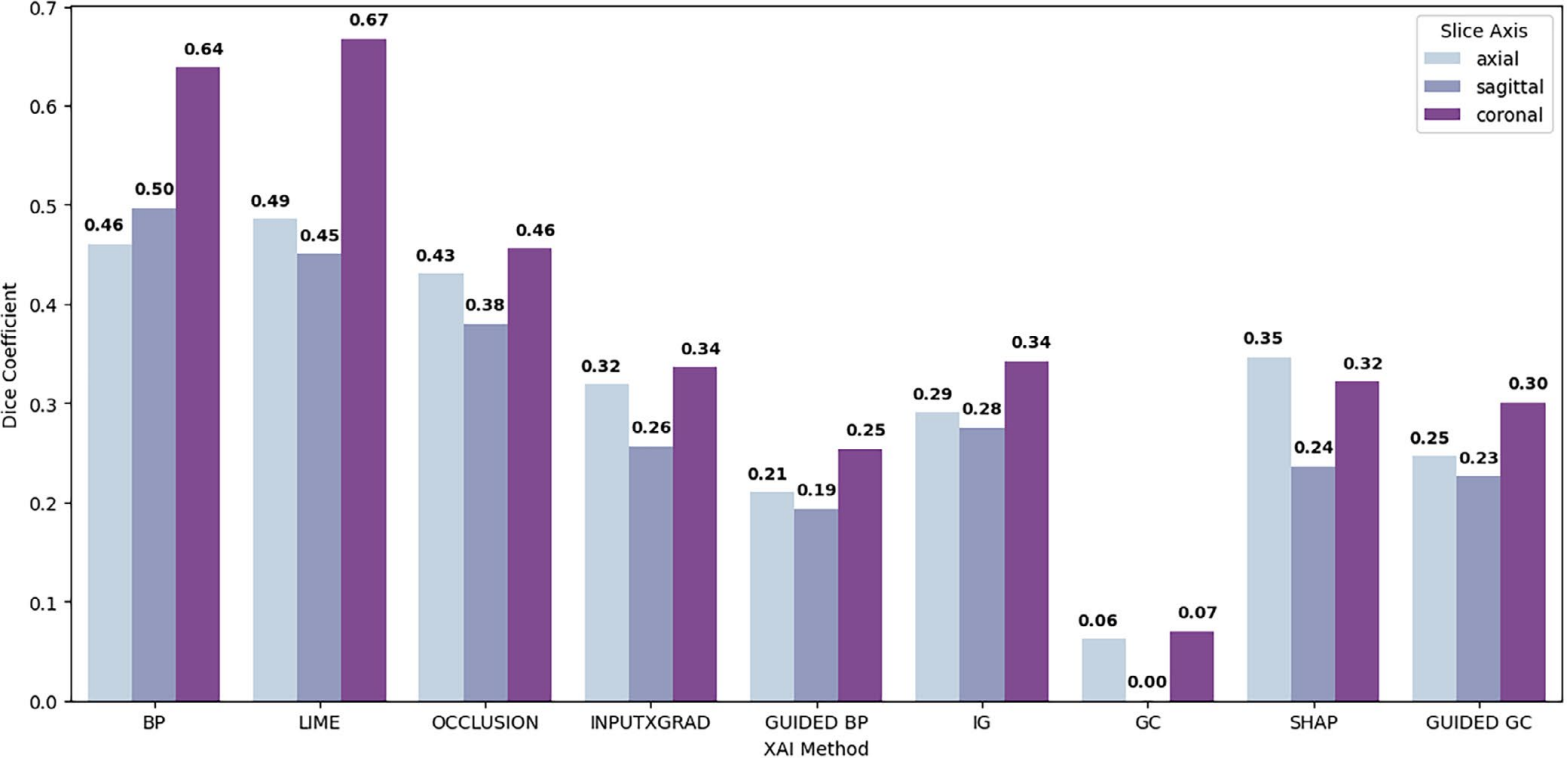
Dice coefficient between binarized saliency maps using the top 10% salient pixels/region in the average ‘AD’ saliency map across nine XAI methods (only methods applicable to both architectures were included). Dice coefficients are given for models trained on three different MRI views: Axial = light blue, sagittal = violet, coronal = purple. BP = backpropagation; GC = GradCAM; LIME = local interpretable model explanations; IG = integrated gradients; InputXGrad = InputxGradient; SHAP = Shapley values.

To quantify consistency across model architectures, we computed the pairwise dice overlap of the most salient regions for axial, coronal and sagittal models. The results shown in Figure 5 suggest that backpropagation and LIME identified the most similar patterns across the CNN and ViT coronal models with Dice coefficients of 0.64 and 0.67, respectively. In contrast, for models trained on sagittal slices, there was little to no overlap in the top 10% salient pixels across architectures.

### 3.3 Saliency Maps for Prognosis Prediction

**FIGURE 6:**
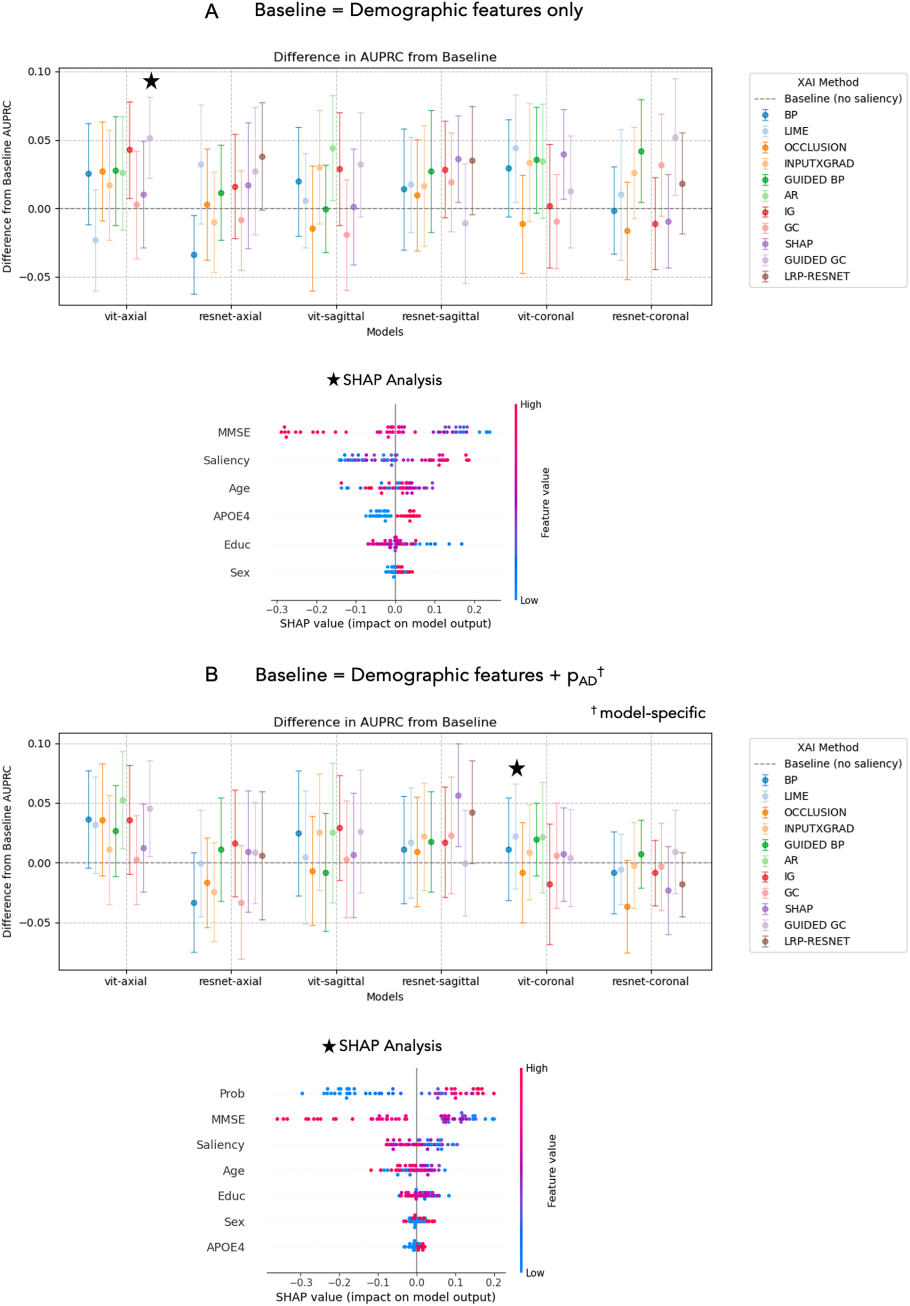
Difference in the area under precision‐recall curve (AUPRC) across models where mean saliency is included in a Random Forest classifier trained and tested on MCI prognosis. We repeated this using 25 random seeds and plot the mean (points) and standard deviation (error bars). ★ denotes the model that yielded the best AUPRC value. SHAP summary plots indicate the contribution of each feature, shown for the optimal model. (A) The difference is between a model trained on clinical and demographic features only. (B) The (relative) difference is between a model trained on clinical‐demographic features and corresponding image‐based probabilities, p_AD_. AR = attention rollout; BP = backpropagation; GC = GradCAM; IG = integrated gradients; InputXGrad = InputxGradient; LIME = local interpretable model explanations; LRP = layer wise relevance propagation; SHAP = (Shap)ley values; ViT = vision transformer.

The results of using data from saliency maps for MCI to AD prognosis prediction are summarised in Figure 6 and Table 4. In Figure 6, we report the increase (mean and standard deviation) in AUPRC compared to a baseline where saliency is not included. For the clinical‐demographic only analysis, the baseline AUPRC was 62.8% and is the same across all models. For baseline with both clinical‐demographic features and imaging‐based probability included, the difference is taken with respect to each model‐specific baseline. SHAP analysis highlights the relative contribution of each feature and is computed for the optimal feature combination. In Table 4 we provide the performance metrics for Random Forest models trained using the optimal feature combinations and the two baseline models. GradCAM mean saliency from the axial ViT model improved the AUPRC by 6.6% compared to a clinical‐demographic baseline. Including both LIME mean saliency and predicted probabilities from the coronal ViT improved AUPRC by a further 16.7%. SHAP analysis revealed that MMSE was the top feature, but saliency ranked highly in both cases.

**TABLE 4:** Random Forest held‐out test performance for MCI prognosis. Models were trained using clinical and demographic features, with the addition of mean saliency and the imaging‐based probability, p_AD_. We report the mean and standard deviation of performance metrics obtained after 25 repeats.

	Prognosis (sMCI (*n* = 25) vs. pMCI (*n* = 31))		
	BACC	AUROC	AUPRC
Clinical‐demo	59.2 ± 0.03	60.3 ± 0.01	62.8 ± 0.03
Clinical‐demo + saliency^a^	62.9 ± 0.02	64.4 ± 0.02	69.4 ± 0.03
Clinical‐demo + p_AD_ ^b^	75.5 ± 0.03	78.5 ± 0.02	84.4 ± 0.02
Clinical‐demo + p_AD_ + saliency^c^	76.6 ± 0.02	79.5 ± 0.02	86.1 ± 0.02

Abbreviations: AUPRC = area‐under‐the‐precision‐recall‐curve; AUROC = area‐under‐the‐receiver‐operator‐curve; BACC = balanced accuracy; Demo = demographics; p_AD_ = imaging‐based predicted probability of dementia.

^a^ Mean guided GradCAM saliency based on the axial ViT model (corresponds to ★ in Figure 6A).

^b^ p_AD_ based on the coronal ViT model (baseline value with respect to ★ in Figure 6B).

^c^ Mean LIME saliency and p_AD_ based on the coronal ViT model (corresponds to ★ in Figure 6B).

## 4 Discussion

We explored the application of XAI to support dementia diagnosis and prognosis with neuroimaging. We show that pretrained ViTs and CNNs can be effectively finetuned for AD classification and predicting MCI conversion. Beyond performance, our findings emphasise the value of XAI to validate model behaviour and inform downstream tasks.

### 4.1 CNNs, ViTs And the Accuracy‐Explainability Trade‐Off

Finetuned 2D models achieved comparable predictive performance to a fully 3D model trained from scratch. This finding is pertinent to considerations around the deployment of AI in resource‐constrained settings where access to high‐resolution 3D data may be limited (Martin, Biondo, et al. 2023). Models also generalised well in held‐out samples from NACC and ADNI, addressing criticisms of existing studies that do not evaluate model transfer to external cohorts (Martin, Townend, et al. 2023; Borchert et al. 2023; Yagis et al. 2021). Overall, 2D ResNet models slightly outperformed ViTs, which is likely due to the ViTs' lack of intrinsic inductive biases and their reliance on patch‐based attention for feature extraction (Raghu et al. 2021). However, this raises questions about the trade‐off between accuracy and interpretability, as ViTs' lack of convolutional filters may make them more transparent.

To mitigate the limitations of single slices, we introduced three strategies for fusing information from orthogonal views. Interestingly, jointly finetuning three ResNet networks allowed us to match the performance of a fully 3D network trained from scratch (+0.1% AUPRC, +2.2% AUROC, −2.75% BACC), suggesting considerable redundancy across the 3D image volume. Our approach aligns with other attempts to leverage ViTs in small datasets such as Alp and colleagues (Alp et al. 2024) who combined an ensemble and a recurrent network to capture inter‐slice dependencies across 50 slices in each plane, achieving accuracies up to 99% on ADNI test data. However, applying fusion strategies can limit explainability, which may be crucial for clinical contexts. Additionally, the difference in performance between the 2D models and 3D model may also be linked to their resolution disparity, for which the 3D model was trained on 96 × 96 × 96 mm volume in contrast to 224 × 224 mm slices. Whilst the primary aim of this study was not to optimise performance, but rather to train models capable of generating meaningful explanations, these results highlight the potential in exploring the compatibility between XAI methods and fusion‐based approaches.

### 4.2 Explainable Artificial Intelligence as a Quality Check

At a high level, model explanations can serve as an initial quality check, to verify whether models focus on relevant regions or exhibit evidence of bias. Notably, to minimize preprocessing we chose not to apply skull stripping, resulting in non‐zero non‐brain MRI intensities which could have introduced a source of bias as observed in related work (Guo et al. 2024). However, the saliency maps in Figure 4 confirmed that the most salient regions lie within the brain. Exceptions were the average GradCAM saliency map, where saliency outside the brain likely reflected artifacts from upsampling or the choice of convolutional layer, and also in some LIME and occlusion saliency maps, which are prone to noise due to their iterative mechanisms (Kindermans et al. 2019). Across most XAI methods we found that models converged upon brain regions known to be relevant to AD such as the hippocampus, cingulate gyrus and ventricular areas. This indicates that in the absence of preprocessing, models were able to disregard background intensities and supports strategies in favour of encouraging robustness through data augmentation over strict harmonisation.

### 4.3 Comparing Explanations Across Models and Methods

Upon visual inspection, gradient‐based techniques such as guided backpropagation, (gradient) SHAP, InputXGradient and Integrated Gradients identified similar patterns for ViT and ResNet models. In line with the comparative framework, this likely reflects that these XAI approaches all rely on learned model weights and differ mainly in their incorporation of the input. Additionally, the convergence of their maps adds validity to the identified most salient regions. In contrast, perturbation‐based methods such as LIME and occlusion produced coarser features but with distinguishable differences on an anatomical, region‐level. However, it is important to consider that perturbation methods are inherently linked to the raw output and are therefore more likely to be sensitive to differences between models (Supporting Information: Figure A3).

To take this further, the results in Figure 5 aim to quantify the agreement of the most salient regions between CNNs and ViTs. LIME, a model‐agnostic XAI technique, produced the most similar explanations for the coronal CNN and ViT models, whilst GradCAM saliency maps generally showed little overlap. In the context of the framework depicted in Figure 1, the convergence of LIME‐based salient regions helps to strengthen the validity of hippocampal features shown in coronal slices. However, this was not consistent across all XAI methods, highlighting the variability of explanations in practice, particularly for medical imaging data where the discriminative features are often more subtle than in natural images. In some cases, the discrepancy may reveal fundamental differences between the two model architectures. For example, GradCAM is a method originally designed for CNNs and despite the use of a model‐agnostic CAM implementation, this may still affect their suitability for ViTs (Selvaraju et al. 2016). This may explain the lack of overlap in GradCAM explanations produced for the ResNets and ViTs. However, these differences may also be indicative of feature redundancy, where different patterns and feature combinations can yield the same prediction. In the context of dementia, where pathology is widespread and neuroanatomical regions are highly correlated, models may choose to focus on unique sets of features, leading to different explanations despite similar performance results. These effects prompt a nuanced view of the scope of XAI and strengthen the argument for employing multiple methods to obtain a more comprehensive understanding of model decisions.

### 4.4 Saliency Maps Enhanced MCI Prognosis

A Random Forest model trained with mean saliency as an additional feature has considerably improved accuracy for prognosis prediction compared to the imaging only models (Figure 6, Table 4). Including mean saliency generally led to an increase in the test AUPRC, especially compared to a model trained only on clinical and demographic features. However, the extent of this improvement varied across XAI methods, which highlights the importance of understanding the assumptions of XAI methods to interpret their outputs accurately (Martin, Townend, et al. 2023). Additionally, in some cases, including mean saliency hindered performance, potentially due to polarizing feature influences or poor‐quality saliency maps. Despite this, our results illustrate how XAI outputs can provide additional information for a downstream classification task beyond traditional clinical markers.

### 4.5 Study Limitations and Future Directions

One limitation of our study is the restriction to 2D models which are often suboptimal compared to 3D approaches. Despite this, the high computational cost of training 3D models and applying certain XAI techniques emphasises the significance of achieving competitive performance with single (or few) slices. Importantly, we re‐emphasise that optimising performance was not the current focus and that further tuning model hyperparameters may alter our results.

A recent study reported superior performance of ViTs over ResNets for dementia classification using 2D MRI slices and incorporating a convolutional patch embedding step (Lyu et al. 2022). This contrasts with our results; however, we note that conducting a fair head‐to‐head comparison of these architectures is not trivial despite our efforts to keep design choices such as training data size and strategies consistent (Bai et al. 2021). Also, whilst ViT‐CNN hybrid approaches have been shown to improve ViT performance in several studies (Lyu et al. 2022; Khatri and Kwon 2024; Zhao et al. 2023; Li et al. 2022), this was not explored here so as to maintain the distinction between a convolution‐free (ViT) and convolution‐based (ResNet) model and thus retain the advantage of ViTs' inherent explainability. Nonetheless, future work could explore extensions to the ViT that are still convolution‐free, such as the Swin transformer (Liu et al. 2021), which may balance performance and interpretability. We also found that models trained on coronal slices performed best, which is likely due to the prominence of the hippocampi in this plane. As such, our approach could be extended by targeting specific slice locations to increase the discriminative ability without requiring the entire 3D volume. The limited availability of pretrained ViT models also restricted us to those pretrained on natural images. In the advent of foundation models and open, medical imaging datasets, further work could investigate the use of neuroimaging‐based pretraining tasks such as brain‐age prediction (Kunanbayev et al. 2024) or sex classification (Dhinagar et al. 2023).

Uncertainty introduces another challenge, as potential misdiagnoses and delays between imaging and clinical assessment may impact the reliability of assigned labels. In the NACC sample, labels were assigned based on clinical assessments within 6 months of the baseline scan; however, ADNI visits are more routinely collected, potentially explaining the increase in performance in external test data. For MCI participants, we used a window of 3 to 4 years for conversion to align closely with ADNI. However, ADNI MCI participants had a higher MMSE compared to NACC on average, indicating a healthier cohort that may be harder to classify. This could explain the lower zero‐shot prognostic recall in ADNI (highest value of 45%) compared to NACC (highest value of 73%).

Our comparison of the XAI outputs remains largely qualitative due to the absence of a ground truth. In Figure 5, we used the Dice coefficient to quantify the agreement between explanations produced for CNNs and ViTs; however, this metric does not always provide an accurate measure of agreement, particularly when used on smaller structures as reported in existing work (Guo et al. 2024; Reinke et al. 2021 ). Existing XAI validation studies have also compared salient regions against ROIs derived from meta‐analyses (Wang et al. 2023; Leonardsen et al. 2024 ), or simulated data (Guo et al. 2024). Whilst these approaches can be useful for assessing group‐level saliency maps or explaining global model behaviour, there remains a need to design strategies to validate explanations at the individual level. Additionally, whilst quantitative analyses provide a systematic way to evaluate the robustness and content of model explanations, this is not sufficient for capturing their practical utility and impact. Our analysis of the use of mean saliency for MCI prognosis aimed to highlight whether useful information can be gained from model explanations; however, to understand the impact of model explanations on trust and clinical decision‐making, further studies focusing on usability and human‐computer interaction will be required.

In this study, the AD and CN group within NACC were not matched for age or sex before training (statistically significantly different, *p* < 0.05). Therefore, when interpreting the outputs of XAI methods, it is important to consider that the salient regions may reflect non‐pathological patterns that influence the model's prediction. For example, ventricular and white matter regions were highlighted in Figure 4, which have been associated with structural brain differences across age and sex in prior research (Gur et al. 1999; Fjell and Walhovd 2010). However, these characteristics are also known correlates of dementia (Gao et al. 1998), such that even within an age‐ and sex‐matched training cohort, these relevant regions would likely appear. Nonetheless, the interplay between correlated features, disease‐specific and otherwise, further emphasises the importance of context and domain knowledge when reviewing model explanations.

From a technical perspective, XAI parameter and processing choices also affect the interpretation of model explanations. For example, the occlusion value is an important parameter, and while we used a default value of 0, this value can carry information in the context of MRI. Similarly, our modified LRP implementation for ResNets relied on predefined rules for specific layers, which were chosen to align with the original implementation (Bach et al. 2015). Exploring alternative configurations may yield different insights and warrants further investigation. Moreover, in our study we applied thresholding, ReLU and intensity scaling to post‐process the saliency maps. These steps are commonly used to mitigate noise and focus on the most important regions in XAI research but can impact subsequent quantitative analysis and interpretation. For example, by applying ReLU, we ignored negative saliency values which can be produced by methods such as LRP. However, as this behaviour is not shared by all XAI methods we chose to focus on positive contributions only. Similarly, data preprocessing choices are also important to consider when analysing model explanations. We chose not to apply skull stripping to the MRI scans. Whilst models were able to focus on relevant brain regions as highlighted in Section 4.2, this decision influences the analysis of the proportion of non‐brain saliency, as some methods like InputXGrad are intrinsically tied to the value of each pixel in the input image. Skull stripping ensures that all non‐brain voxels are nullified and as such would have had a direct impact on these results. Future research would benefit from systematically exploring the impact of image processing on model explanations and investigating these effects further.

### 4.6 Implications for Clinical Translation

This study serves as a starting point for understanding the applicability of XAI to dementia prediction tools. We suggest that due to the high variability of XAI outputs, future work should consider multiple methods to identify converging patterns and detect potential biases. We also emphasize that the performance advantages of CNNs should be weighed against the potential transparency of more interpretable models such as ViTs, particularly in clinical applications. Whilst we found that models converged on the most salient regions after thresholding, the granularity of salient regions varied markedly across methods. These differences may become significant depending on the task and intended use of model explanations (Chen et al. 2022). For example, occlusion and LIME outputs were more brain‐like in size and shape, which may be more useful for visually confirming known regions of interest. In contrast, techniques such as Integrated Gradients can highlight pixel‐level saliency and may be better suited to applications where local accuracy is important such as (regional) segmentation or voxel‐based analyses.

## 5 Conclusion

This work presents a comprehensive application of current XAI approaches to two popular deep learning architectures for dementia prediction. We demonstrate that transfer learning can be used to leverage pretrained models, exploring strategies for fusing information across multiple views. Despite their limitations, XAI methods provide useful information, serving as a tool to understand what information the model is focusing on with potential to derive new features. We also propose a framework for comparing explanations across XAI techniques and models to reveal both contrasting and converging evidence of salient features. Model explanations may also contain diagnostically useful information as explored through the addition of a saliency‐derived feature for predicting future dementia diagnoses. These findings open the door for further work in designing quantitative, context‐specific validation frameworks and assessing the clinical utility of model explanations.

## Author Contributions

**Sophie A. Martin:** conceptualisation, methodology, software, validation, investigation, data curation, visualisation and writing – original draft. **An Zhao:** methodology. **Jiongqi Qu:** data curation. **Phoebe Imms:** methodology. **Andrei Irimia:** methodology. **Frederik Barkhof:** conceptualisation, methodology, supervision. **James H. Cole:** conceptualisation, methodology, supervision. All authors read, reviewed and approved the final manuscript.

## Funding

This work was supported by the EPSRC (EP/S021930/1); NIHR; Alzheimer's Research UK (ARUK‐PPG2023B‐016); National Institute on Aging (P30AG017265); National Institutes of Health (2T32AG000037‐46, R01 NS100973, RF1 AG082201, R01 AG 079957, and U19AG024904); Department of Defense (under contract W81XWH‐18‐1‐0413).

## Consent

The authors have nothing to report.

## Conflicts of Interest

Frederik Barkhof reports board membership from Neurology, board membership from Radiology, board membership from Neuroradiology, personal fees from Springer, personal fees from Biogen, grants from Roche, grants from Merck, grants from Biogen, personal fees from IXICO, grants from European Innovative Medicines Initiative, grants from GE Healthcare, grants from the UK Multiple Sclerosis Society, grants from the Dutch Multiple Sclerosis Research Foundation, grants from the National Institute for Health and Care Research, personal fees from Combinostics, and personal fees from Prothena, outside the submitted work; and is co‐founder and stock owner of Queen Square Analytics. The other authors declare no conflicts of interest.

## Supporting information


**Data S1:** hbm70456‐sup‐0001‐supinfo.docx.

## Data Availability

The data that support the findings of this study are available from the National Alzheimer's Coordinating Centre and Alzheimer's Disease Neuroimaging Initiative. Restrictions apply to the availability of these data, which were used under license for this study. Data are available from https://naccdata.org and https://adni.loni.usc.edu with the permission of the National Alzheimer's Coordinating Centre and Alzheimer's Disease Neuroimaging Initiative respectively.
