# Supplementary material for: Investigating the Utility of Explainable Artificial Intelligence for Neuroimaging‐Based Dementia Diagnosis and Prognosis
Source: Hum Brain Mapp. 2026 Feb 2;47(2):e70456. doi: 10.1002/hbm.70456 (PMC12862880; doi:10.1002/hbm.70456)
Supplement: Supplementary file 1 — Data S1: hbm70456‐sup‐0001‐supinfo.docx. [file HBM-47-e70456-s001.docx]

**Supplementary Material**

**A1 –** Participant filtering and data processing pipeline for NACC

Images were downloaded from the NACC June 2022 freeze (prior to SCAN initiative availability). Therefore, all imaging data is voluntarily uploaded by the individual ADRCs and are not subject to routine preprocessing or quality control checks as in the ADNI database. To remove poor quality scans, we utilised visual assessment and MRIqc image quality metrics to exclude images with any outliers based on CJV, SNR or CNR values. Additionally, participants were removed due to download issues, incorrect file header information and a lack of available clinical diagnosis of CN, MCI or ADD within a 6-month time-period from their baseline scan. Some participants who received follow-up diagnosis were classified as reverters where their baseline diagnosis of MCI or AD later improved (e.g., MCI to CN, AD to MCI, or AD to CN). These participants were also removed from our analysis.


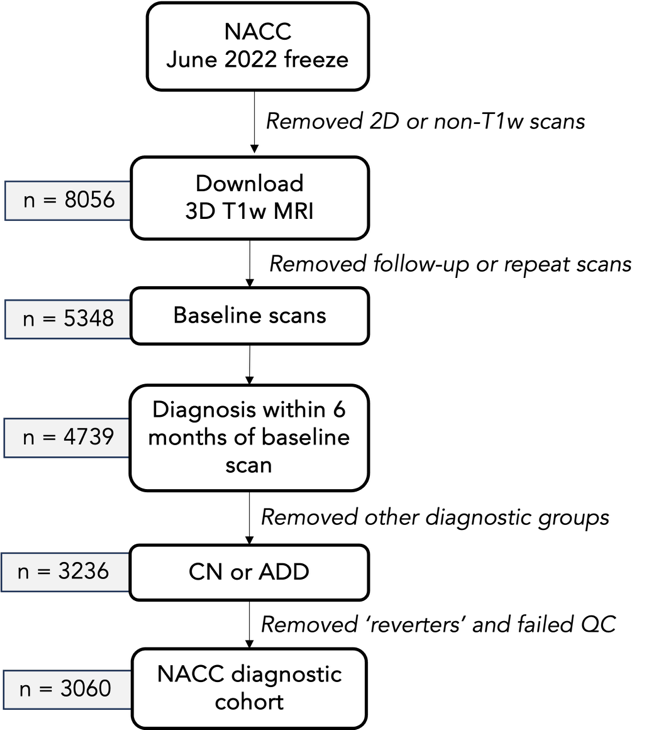


Figure A1. Data flow for NACC data processing.

**A2 –** MRIqc image quality metrics in NACC and ADNI


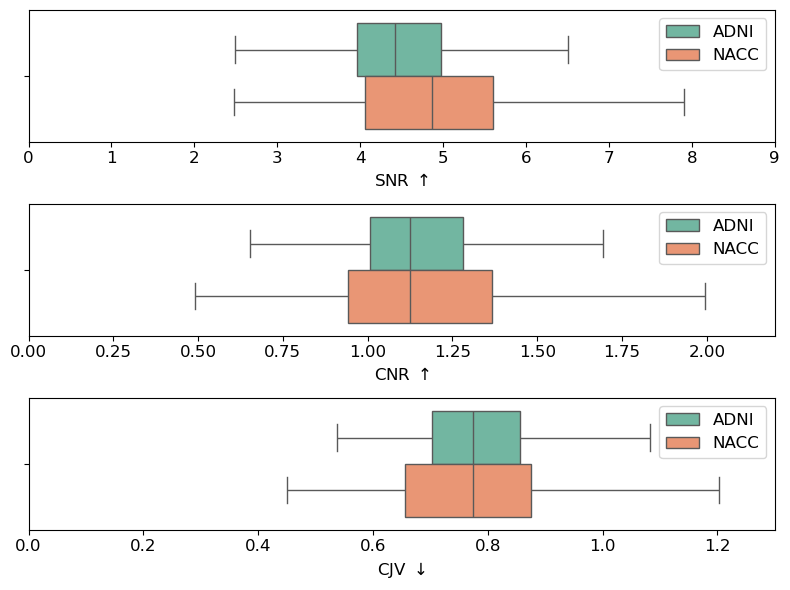


Figure A2. MRIqc derived image-quality metrics for NACC and ADNI 3D T1-weighted MRI data (AD and CN individuals only, after removal of outliers). Note, the two datasets have different sample sizes.

**A3 –** CNN and ViT predicted probabilities


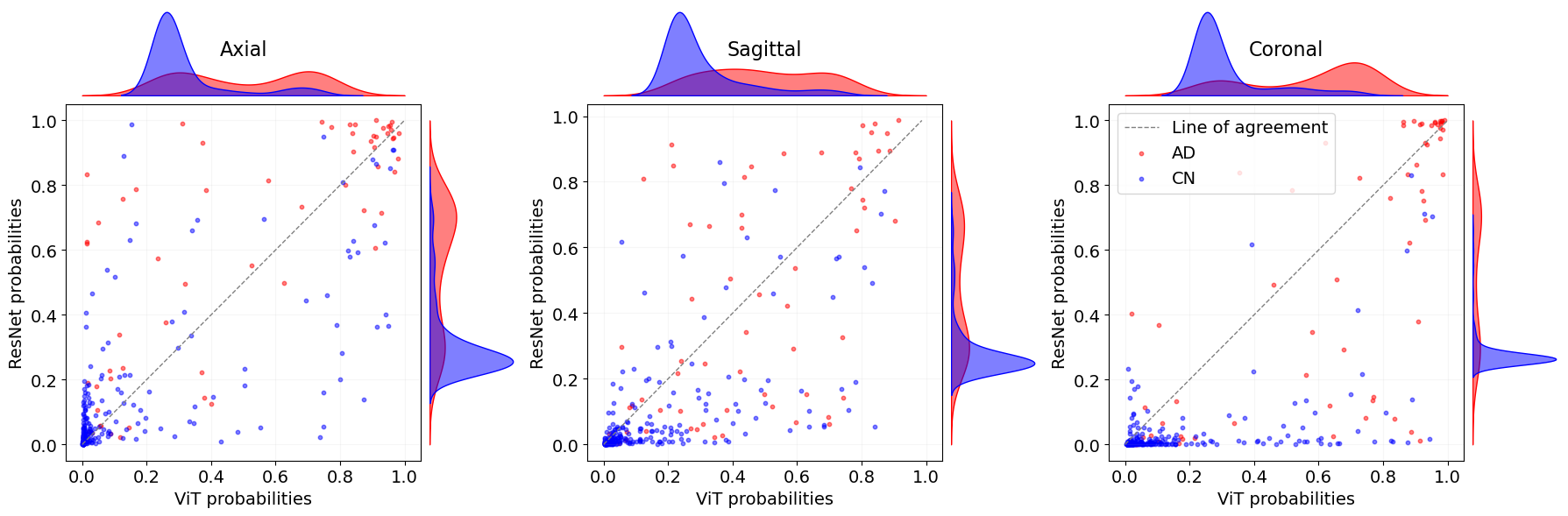


Figure A3. Scatter plots (with kernel density estimation) of single-slice model test predictions for AD dementia classification across both architectures.

**A4 –** Proportion (%) of non-brain saliency across XAI methods and models


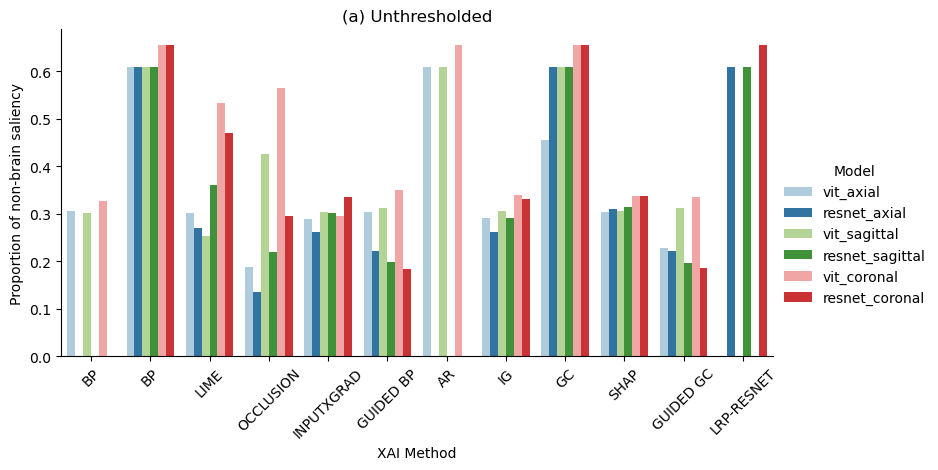


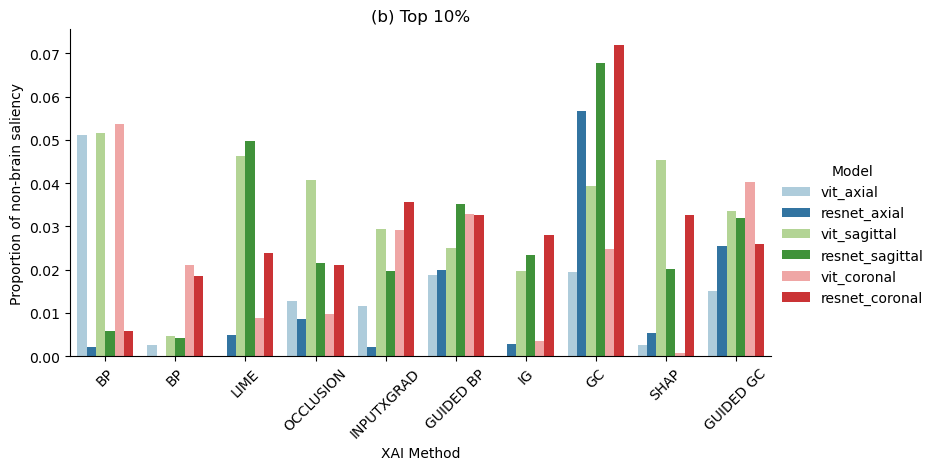


Figure A4. Proportion (%) of non-brain saliency across XAI methods and slice-wise models: (a) unthresholded (b) thresholded to the top 10% intensity. Abbreviations: ViT = vision transformer, LRP = layer wise relevance propagation, AR = attention rollout, BP = backpropagation, IG = integrated gradients, InputXGrad = InputxGradient, CAM = class activation mapping, LIME = local interpretable model explanations, SHAP = (Shap)ley values.

**A5 –** Vision transformer and ResNet architecture details and training hyperparameters

|  | **Vision Transformer** | **ResNet** |
| --- | --- | --- |
| Architecture | *vit-tiny-patch16-224* | *resnet18d_21k* |
| # Params | 5.7M | 11.7M |
| Fixed parameters (different from default) | in_chans: 1  attn_dropout: 0.1  num_classes: 1 | in_chans: 1  num_classes: 1 |
| Hyperparameter search (optimal values shown in **bold)** | Initial learning rate: [1e-10, **1e-4**, 1e-2]  Scheduler: [‘reduce_on_plateau’, **None**]  Optimiser: [‘sgd’, **‘adamw’**]  Dropout: [0, 0.1, **0.5**] Momentum: [**0.9**, 0.5, 0.99] Weight decay: [0, 1e-2, **1e-4**] | Initial learning rate: [1e-10, 1e-4, **1e-2**]  Scheduler: [‘**reduce_on_plateau’**, None]  Optimiser: [**‘sgd’**, ‘adamw’]  Dropout: [**0**, 0.1, 0.5] Momentum: [0.9, 0.5, **0.99**] Weight decay: [0, 1e-2, **1e-4**] |

Table A5. Details on the model architectures used for the ViT and ResNet-18 and hyperparameter search values (optimised values are shown in **bold**). We performed a grid search hyperparameter search for axial slices models using 20% of the training data for validation, and kept these parameters fixed for the coronal, sagittal, and 3D models. Pretrained models were obtained from the timm library.

**A6 –** MLP hyperparameters for late fusion model

| **Parameter** | **Search values** | **Method** |
| --- | --- | --- |
| **hidden_size** | [1, **3**, 5] | Grid search |
| **num_layers** | [0,**1**,3] | Grid search |
| **learning_rate** | [0.0001, 0.001, **0.01**, 0.1] | Random choice |
| **batch_size** | [8,**32**,64] | Random choice |

Table A6. Multi-layer perceptron hyperparameters and values that were explored over 10 trials to optimise the triplanar ensemble (late) model. The optimal model was chosen based on the validation AUPRC (20% subset of training as before) using the ViT model’s predictions and kept fixed for the ResNet triplanar-late model. Optimised values are shown in **bold**.

**A7 –** Random Forest hyperparameters and search values

| **Parameter** | **Search values** |
| --- | --- |
| **max_depth** | [None, 2, 5,10] |
| **min_samples_leaf** | [1,3,5,10] |
| **min_samples_split** | [2,5,10] |
| **n_estimators** | [1,3,10,50] |
| **criterion** | [‘gini’, ‘entropy’, ‘log_loss’] |
| **class_weight** | [‘balanced’, None] |

Table A7. Random Forest (RF) hyperparameters and values that were searched during 5-fold CV grid search optimisation using scikit-learn. Optimisation was conducted for all RF models independently.

**A8** – 5-fold cross-validation performance

Table A8. 5-fold cross-validation was performed to assess the stability of the finetuned models’ performance.

|  | **5-Fold Cross Validation Diagnostic Performance** | | | | | |
| --- | --- | --- | --- | --- | --- | --- |
|  | Vision Transformer | | | ResNet | | |
|  | BACC | AUROC | AUPRC | BACC | AUROC | AUPRC |
| **Axial** | 72.5±5.19 | 86.6±1.41 | 68.6±4.20 | 80.6±1.34 | 89.0±1.95 | 76.2±2.34 |
| **Coronal** | 79.4±3.26 | 90.9±1.80 | 79.6±2.28 | 83.1±3.39 | 93.3±1.39 | 85.1±1.89 |
| **Sagittal** | 69.9±4.24 | 84.3±0.91 | 64.4±4.06 | 72.2±2.09 | 88.6±1.26 | 72.0±3.51 |
